# Functional Nanocellulose, Alginate and Chitosan Nanocomposites Designed as Active Film Packaging Materials

**DOI:** 10.3390/polym13152523

**Published:** 2021-07-30

**Authors:** Gregor Lavrič, Ana Oberlintner, Inese Filipova, Uroš Novak, Blaž Likozar, Urška Vrabič-Brodnjak

**Affiliations:** 1 Pulp and Paper Institute, Bogišićeva Ulica 8, 1000 Ljubljana, Slovenia; gregor.lavric@icp-lj.si; 2 National Institute of Chemistry, Hajdrihova 19, 1000 Ljubljana, Slovenia; ana.oberlintner@ki.si (A.O.); uros.novak@ki.si (U.N.); blaz.likozar@ki.si (B.L.); 3 Jožef Stefan International Postgraduate School, Jamova Cesta 39, 1000 Ljubljana, Slovenia; 4 Latvian State Institute of Wood Chemistry, Dzerbenes Street 27, LV-1006 Riga, Latvia; inese.filipova@kki.lv; 5 Department of Textiles, Graphic Arts and Design, Faculty of Natural Sciences and Engineering, University of Ljubljana, Snežniška 5, 1000 Ljubljana, Slovenia

**Keywords:** carbohydrate, polysaccharide, nanocellulose, alginate, chitosan, film packaging material, functional active design, biomass-derived biomaterial nanocomposites, oxygen/air/water barrier properties, bio-based biopolymer composites for food

## Abstract

The aim of the study was to characterize and compare films made of cellulose nanocrystals (CNC), nano-fibrils (CNF), and bacterial nanocellulose (BNC) in combination with chitosan and alginate in terms of applicability for potential food packaging applications. In total, 25 different formulations were made and evaluated, and seven biopolymer films with the best mechanical performance (tensile strength, strain)—alginate, alginate with 5% CNC, chitosan, chitosan with 3% CNC, BNC with and without glycerol, and CNF with glycerol—were selected and investigated regarding morphology (SEM), density, contact angle, surface energy, water absorption, and oxygen and water barrier properties. Studies revealed that polysaccharide-based films with added CNC are the most suitable for packaging purposes, and better dispersing of nanocellulose in chitosan than in alginate was observed. Results showed an increase in hydrophobicity (increase of contact angle and reduced moisture absorption) of chitosan and alginate films with the addition of CNC, and chitosan with 3% CNC had the highest contact angle, 108 ± 2, and 15% lower moisture absorption compared to pure chitosan. Overall, the ability of nanocellulose additives to preserve the structure and function of chitosan and alginate materials in a humid environment was convincingly demonstrated. Barrier properties were improved by combining the biopolymers, and water vapor transmission rate (WVTR) was reduced by 15–45% and oxygen permeability (OTR) up to 45% by adding nanocellulose compared to single biopolymer formulations. It was concluded that with a good oxygen barrier, a water barrier that is comparable to PLA, and good mechanical properties, biopolymer films would be a good alternative to conventional plastic packaging used for ready-to-eat foods with short storage time.

## 1. Introduction

According to estimates by the United Nations Joint Group of Experts on the Scientific Aspects of Marine Pollution (GESAMP), between 70 and 95% of waste will join millions of tons already present in seas, lakes, air, rivers, groundwater, crop fields, landfills, and cities. Most of this waste is coming from the traditional commercial food packaging materials with a petroleum-based origin such as polyethylene (PE), polypropylene (PP), and polystyrene (PS). Researchers have estimated that 31.9 million tonnes of mismanaged plastic waste enter the environment every year, with 4.8–12.7 million tonnes going into the oceans and significant quantities contaminating terrestrial ecosystems [1,2]. Still, it is expected that the demand for plastics will continue to grow in the future to enable resource-efficient products needed by society. One of the establishing trends is in designing mindful products from sources including recycled chemicals and renewable raw materials, and using processes powered by renewable energy in striving to establish an efficient circular economy [3]. To be in line with the UN sustainable development goals and sustainable plastic strategy, the global plastics industry’s shift from a manufacturing system based predominantly on fossil fuels to sustainable and affordable alternatives is being envisioned [4,5].

Packaging materials are an essential part of product processing, therefore the number of investigations on the development and use of new alternatives has increased in recent times. That the transition towards non-virgin petrochemical and bio-based raw materials as alternative feedstocks should include recycled chemicals from plastics waste, sustainable biomass, industrial wastes such as CO_2_, and modified biopolymers such as cellulose or starch, is suggested mainly due to the interest in minimizing the environmental impact caused using synthetic packaging materials. One of the most desired features expected in packaging is the capability of decomposing into carbon dioxide, methane, water, inorganic compounds, or biomass, the dominant mechanism of decomposition being the enzymatic action of microorganisms and that the resulting products can be obtained and measured in a period of a certain time [6]. The materials used to make biodegradable packaging can be biopolymers of natural origin (alginate, starch, gelatine, collagen, proteins, chitosan, (nano)cellulose, pectin) or of synthetic origins, such as polylactic acid, polycaprolactone, and polyvinyl alcohol [7,8]. Especially the materials of natural origin have recently (re)gained popularity, due to their special properties, which in many industries can become an alternative for fossil fuel-based plastic. However, high production costs, low performance, and not less important, ethical implications, still hinder the market penetration of plastics-free alternatives so far. One of the currently underutilized sources of feedstock for bio-based polymers can be found in the side streams of both agricultural and forest feedstock, which are a good source [9]. Cellulose, which exists in the lignocellulosic biomass, is the most abundant polysaccharide present in nature. It is encapsulated by lignin and hemicellulose and produces a linear polysaccharide with nanometre diameter by repeating the connection of β-d-glucose [10]. Cellulose nanofibrils (CNFs) or cellulose nanocrystals (CNCs) can be isolated from wood and other plant sources by partial disruption of their natural structures, which is usually achieved using chemical and/or mechanical treatments. Besides plants, some bacteria naturally produce cellulose microfibrils, which are referred to as bacterial nanocelluloses (BNCs) [11]. These three types of nanocelluloses (CNCs, CNFs, and BNCs) have different morphologies (sizes and shapes) depending on their biological origin and the processes used to isolate them [12,13]. Unlike the rigidity of the CNC, the CNF is flexible. This is mainly because the structure of CNF is an individual or aggregated soft and long chain, which is formed by alternately connecting crystalline regions and amorphous regions to each other [9,10,11].

The second most abundant natural biopolymer is chitin, the crucial structural biopolymer of crustaceans’ exoskeletons, whereby its content varies not only between different sources but also between different species [9]. These types of polysaccharides have previously mostly been utilized and studied for biomedical applications [14]. However, in the search for new carbon-neutral renewable resources using the biorefinery approaches, turning cast-off shells into nitrogen-rich chemicals would benefit economies and the environment [15]. Other largely exploited sources for biopolymers are macroalgae, which are the rich source of indigestible polysaccharides that are commonly produced by and refined from various brown seaweed and can be developed into active food packaging materials [16,17]. In the recent review from Zhang et. al., the effect of the incorporation of CNC on the film’s characteristics, including thickness, optical properties, barrier properties, water sensitivity, mechanical properties, antioxidant properties, and antimicrobial properties have been presented [18]. The main advantage of using cellulose nanoparticles as a reinforcing part in the film is most addressed by the “tortuous theory”, where the cellulose nanoparticles, due to their size, form a denser microstructure, which mostly leads to an increase in the mechanical strength of the composite (bio)material. Moreover, cellulose nanoparticles act as a physical barrier structure in the composite film materials leading to reducing the movement of gas molecules through the film. The films with a higher degree of nanocellulose fibrillation absorbed more water, and had the higher contact angles for glycerol and lower contact angles for water [19]. Recently, many studies have shown that the incorporation of cellulose nanomaterials as an additive could improve the performance of the food packaging films [20,21,22]. Further advances in nanocellulose research in biopolymers film are quite promising for active packaging applications, including the controlled release packaging and responsive packaging [22,23,24,25].

This study aimed to evaluate and compare the effect of the CNC, CNF, and BNC cellulose nanomaterial, where the first two were obtained from lignocellulosic biomass and the last one from bacterial origin. Altogether, 25 combinations of biopolymer films using chitosan, alginate, or nanocelluloses as a single component or in different combinations were prepared. Based on the determined mechanical properties in the first part of the study, the best film in terms of mechanical properties and physical appearance were selected and further characterized for density, contact angle, surface energy, water absorption, and morphological examination with SEM, oxygen, and water barrier properties. Finally, some suggestions and challenges of potential new sustainable packaging that need a further improvement/focus to commercially exploit this (nano) material renewable bioresource for packaging application.

## 2. Materials and Methods

### 2.1. Materials

High molecular weight (Mw = 310,000–375,000 Da) chitosan (<75% deacetylated), sodium alginate and 85 wt% lactic acid were purchased from Sigma Aldrich (Steinheim, Germany) and glycerol was purchased from Pharmacem Sušnik (Ljubljana, Slovenia). Nanocellulose materials were produced as presented in the sections below. 

#### 2.1.1. CNF Production

For CNF production, bleached hardwood Kraft pulp (kindly provided by Metsä Fibre, Ainekoski, Finland) was oxidized at 70 °C for 4 h in APS (ammonium persulfate) solution (APS: fibers amount ratio 5:1) with continuous stirring. Oxidation was stopped by cooling the mixture to 15 °C, treated fibres were washed until neutral and kept at 4 °C. Oxidized cellulose fibres were then suspended in water (1.5% *w/w*), sonicated (ultrasonic homogenizer SONIC-650W, MRC Ltd., Holon, Israel) for 15 min (90% power, 9 s on, 1 s off), and then processed in microfluidizer (LM20, Microfluidics, Quadro Engineering, Waterloo, ON, Canada); the first 3 times through 200 µm ceramic chamber H30Z, then through 100 µm diamond chamber H10Z at 300–600–900–1500 bar, three passes at each pressure, followed by 6 passes at 2000 bar. Sample was cooled in an ice bath during the treatment. Semi-transparent viscous 1.5% *w/w* solution was obtained and kept at 4 °C until used. The yield of CNF reached ~80% from the initial amount of pulp.

#### 2.1.2. BNC Production

The raw material for BNC production was a cellulose-rich bio-film formed after acetic fermentation of apple juice [26] and was obtained from a local vinegar producer. Bio-film was thermo-mechanically treated as described in the article by Lavrič, Medvešček, and Skočaj [27]. Treatment separated the individual nanofibrils, resulting in the formation of a homogeneous semi-transparent 0.5% *w/w* BNC gel. The yield of BNC from the initial solution (mother of vinegar) was ~87%. The rest were removed impurities (mainly brownish-colored particles of the apple pulp that had served as the raw material for the vinegar production).

#### 2.1.3. CNC Production

CNC was prepared in accordance with the procedure described by Kunaver, Anžlovar, and Žagar in 2016 [28]. The liquefaction reaction, using glycols and mild acid catalysis (methane sulphonic acid), was applied to eucalyptus wood. The process contains four steps: the milling, glycolysis reaction, centrifugation, and final rinsing with an organic solvent. The yield of CNC was 63 ± 8.5% and the final product was a stable, highly concentrated CNC suspension in water, which was diluted to 1.5% *w/w* before being used in film-forming solutions.

### 2.2. Films Preparation

Films were prepared with different polymer matrices: chitosan, alginate, and nanocellulose. Protocol commonly used in similar studies, e.g., [29], for dissolution, blending, and casting of alginate, chitosan, and nanocellulose was used. Chitosan and alginate film-forming solutions were prepared at concentrations 1.5% *w/w* by dissolving chitosan in 1 wt% aqueous solution of lactic acid and glycerol, and by dissolving sodium alginate in ultrapure water. Dissolution was realized for approximately 24 h under constant stirring with a magnetic stirrer (Ika, Staufen, Germany). The mixtures were then vacuum filtered through 4 layers of medical gauze to eliminate impurities. For chitosan and alginate films with nanocellulose additives, CNC, CNF, or BNC in amounts of 3 or 5 *w/w*% with respect to main biopolymer were added to prepared chitosan and alginate solutions and then homogenized with UltraTurrax (Ika, Staufen, Germany). To eliminate the air bubbles in the film, the mixtures were left overnight. For nanocellulose films, corresponding solutions BNC 0.5% *w/w*, CNF 1.5% *w/w* (or diluted to 0.5–1.0% *w/w* if viscosity was too high) and CNC 1.5% *w/w* were used. Glycerol was used as a plasticizer in some types of films and was added in the amount of 30 wt% with respect to the main biopolymer. The casting volumes of FFS were chosen in a range of 50–100 mL depending on dry mass of polymers in different solutions used and respecting the requisite final film casting weight, which was 47 ± 7 g m^−2^. All films were casted into 12 × 12 cm^2^ polyurethane petri dishes and dried under constant airflow in a laminar flow hood (Microbium d.o.o, Ljubljana, Slovenia) at room temperature and RH 40% for 48 h. In the case of nanocellulose films, a silicon pad was placed on the bottom of Petri dishes to prevent sticking. 

### 2.3. Tensile Properties

Tensile properties of films were determined in accordance with ASTM D 882, using the tensile testing machine Zwick Roell Z010 equipped with 20 N measuring cell (Class 0.5, ISO 7500-1) and the testing software testXpert (Version II V3.2, Zwick GmbH & Co. KG, Ulm, Germany). Samples with a width of 15 mm were tested at 10 mm/min testing speed. The clamping length was set to 70 mm. Testing took place at 23 °C and 50% RH. Samples were exposed to these conditions 48 h before testing.

### 2.4. Water Contact Angle

Film was cut into pieces of approximately 2 × 3 cm^2^ in size and placed onto the microscope glass. Contact angles were measured with water, employing the sessile drop method with Tensiometer Theta T200 (Biolin Scientific, Darmstadt, Germany). The measurements were done in triplicates.

### 2.5. Film Density

Films were cut into pieces of 3 × 3 cm^2^ in size. The thickness was measured with ABS Digital Thickness Gauge (Mitutoyo, Japan) on three different parts of the film and then weighed on an analytical scale. The density was calculated through the Equation (1):

(1) $$\mathrm{Density}=\frac{m}{d\;\cdot S}\;\left(g\cdot {\mathrm{cm}}^{-3}\right)$$

where m is the mass of the tested sample, d is the thickness of the film in cm, and S is the area of the sample. All measurements were done in triplicates. 

### 2.6. Scanning Electron Microscope (SEM)

Film surfaces, as well as pure nanocellulose, were investigated under vacuum conditions by SEM SUPRA 35VP (Carl Zeiss, Jena, Germany). A small amount (approx. 50 mg) of CNC, BNC, and CNF were solvent exchanged to acetone through successive centrifugation steps and then placed onto a piece of microscope glass over a heating plate. This ensures quick evaporation of the solvent and prevents aggregation of nanocellulose. Before analysis, the samples were coated with 10 nm of gold. The size of nanocellulose particles was measured using ImageJ software (Version 1.52, LOCI, University of Wisconsin, Madison, WI, USA) on at least 10 different points. Films were placed on graphite tape before analysis. The magnification of all samples was 10,000×.

### 2.7. Water Vapour Transmission Rate (WVTR) and Water Vapor Permeability (WVP) Determination

Water vapor transmission rate was determined according to the principles of the ISO 2528:2018 standard at 23 °C and 50% RH. Since the hot wax could damage the films during the sample preparation (according to standard procedure), special vessels with a double-sided seal and a system of screws were used to perform the measurements.

(2) $$\mathrm{WVTR}=\frac{\Delta m}{A\cdot t}\;\left(g\cdot {\;\mathrm{cm}}^{-1}\cdot {\mathrm{day}}^{-1}\right)$$

where A is tested area in cm^2^, t time after 24 h of testing, and Δm the mass difference of tested sample. 

Based on WVTR, WVP values were calculated. Calculations were done according to ASTM E96, described by Equation (3):

(3) $$\mathrm{WVP}=\frac{\mathrm{WVTR}}{S\;\left(R_{1}-R_{2}\right)}\;\left(g\cdot {\;m}^{-1}\cdot s^{-1}{\;\mathrm{Pa}}^{-1}\right)$$

where WVTR is calculated through Equation (2), S is the saturation vapour pressure at test temperature (21.068 mmHg at 23 °C), R_1_ is the relative humidity in the environment (50%), and R_2_ is the relative humidity in the test tube (0%).

### 2.8. Oxygen Permeability (OTR)

Oxygen permeability of samples was determined in accordance with ISO-2:2003 at 23 °C and 50% RH using Labthink Perme OX2/230 device (Labthink, Boston, MA, USA). 

### 2.9. Moisture Absorption

Moisture absorption was measured modifying the method proposed by Soni et al. [30]. Films were cut in pieces with dimensions 1 × 3 cm and conditioned at 0% RH (relative humidity) for 24 h. Film samples were then weighted and placed at 85% RH for 24 h. The relative humidity was created with a saturated solution of potassium chloride at room temperature. The samples were weighed, and the moisture absorption was calculated using the Equation (4):

(4) $$\mathrm{Moisture}\;\mathrm{absorption}\;\left(\%\right)=\frac{W_{85}-W_{0}}{W_{0}}\times 100$$

where W_85_ is the weight of the sample after 24 h at 85% RH and W_0_ is the initial weight of the sample after conditioning at 0% RH. Four replicate measurements were taken for each film.

### 2.10. Statistical Analysis

Statistical analysis was done using the one-way ANOVA with the confidence level of 95% (*p* < 0.05) in conjunction with Tukey’s honestly significant difference post-hoc test. All experiments were done in a minimum of five parallels and the results were expressed as the mean ± standard deviation.

## 3. Results and Discussion

### 3.1. Tensile Properties

**Figure 1:**
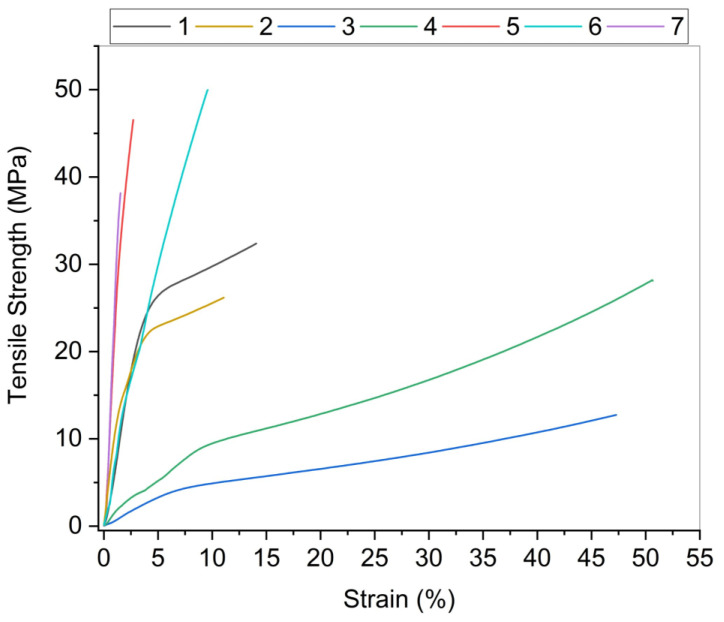
Tensile strength with the respective strain for nanocellulose, alginate, and chitosan nanocomposites. The composition of the films 1–7 are described in Table 1.

Altogether, 25 combinations of biopolymer films using chitosan, alginate, or nanocellulose (NC) as a single component or in different combinations were prepared; however, selection of samples for further investigation was made, based on preliminary evaluation, which was based on the appearance of dry films—integrity, surface properties, visual appearance of film homogeneity, presence of cracks, performance during film handling—the possibility of peeling of the casting dish, and appropriateness for testing (Table S1). Since tensile properties are one of the basic criteria for packaging materials, selection was based also on the results of measured tensile strength (TS) and strain at break (E) of all films, when it was technically possible to perform measurements. Different film compositions showed significantly different results (all the results are given as Supplementary Files), for instance, alginate-based films showed TS from 11.7 ± 0.7 to 42.6 ± 3.6 MPa and chitosan films showed TS from 14.0 ± 2.2 to 30.9 ± 2.2 MPa depending on the amount and type of NC added to the main biopolymer. In the case of chitosan, the addition of any NC type additive improved the mechanical strength of the film; however, in the case of alginate films, the impact depended on the type and amount of NC and was negative in most cases when CNF or BNC was added. As a result of preliminary evaluation, seven films were selected for further investigation: alginate, alginate +5% CNC, chitosan, chitosan +3% CNC, BNC with and without glycerol, and CNF with glycerol. Their properties are Table 1 and Figure 1.

As it was said above, tensile properties are one of the basic criteria for packaging materials. Namely, the mechanical behaviour of packaging films is a very important property of the film to maintain its authenticity and to withstand the environmental impact during the packaging application. The TS and E at break were determined for all film samples. The TS determines the maximum load that can be sustained per cross-sectional area of the film. Strain at break shows the extension of the film, e.g., the flexibility that can be stretched before the breaking point. These characteristics support the correlation of the mechanical properties of films with their compositions and chemical structures. Samples with chitosan and alginate, with the addition of CNC, showed an increase in both TS and E. The average tensile strength of pure CNC films is about 63 MPa, as reported in the literature [31,32]. As expected, the mechanical properties were influenced by the addition of CNC. Films based on alginate exhibited TS of 40 MPa, which increased by 12% upon the addition of 5% CNC. According to the measurement results, the alginate films of all samples showed the best TS/E ratio. Huq et al. [33] reported that the high TS of the alginate-based bio nanocomposite films is due to a good interfacial interaction between the nanofillers and the alginate-based matrix due to similar polysaccharide structures of cellulose and alginate, which was also confirmed on our samples [33]. The largest increase was found in chitosan film, where the addition of CNC improved strength by 120%. The same increase in E was found in both samples (alginate for 30% and chitosan for 6%). It is known from the literature that CNC has a large length/diameter ratio and very good tensile properties. Our analysis confirmed that there are interactions between CNC and chitosan molecules, such as electrostatic association and hydrogen bonding, which create an interactive network and improve overall tensile properties [34]. Cellulose based films have low flexibility, plasticizers should be added to improve this mechanical property and to facilitate the handling of these biopolymers’ films. The most used plasticizer is glycerol due to its stability and compatibility with hydrophilic biopolymer chains [35]. The results have shown that bacterial nanocellulose films (BNC) have the highest TS (60.1 MPa) but a lower E (4.2%). The addition of glycerol changed the properties of the film made of BNC. Namely, the plasticization of BNC with glycerol, which reduced the strength of the hydrogen bonds between adjacent cellulose chains, changed the TS of the film. The TS decreased by 11.6%. At the same time the addition of glycerol increased the E values by about 145% (from 4.2 to 10.3%). According to the results obtained, it is predicted that the moisture absorbed into the matrix of the film had a plasticizing effect. As a result, the TS decreased and E increased by weakening the intermolecular forces, thus increasing the space between the polymers and reducing the crystallinity [36,37]. Overall, by improving the strain of rather rigid films, the glycerol improved their suitability for packaging materials.

CNF were produced from hardwood Kraft pulp by a mechanical process with previous chemical oxidation with APS, as described previously. The results showed that the fibrils were shorter and thinner compared to CNF produced by the TEMPO process [37], which is caused by the fibre cleaving effect of persulfate. Similar reinforcement properties for APS and TEMPO oxidized CNF have been proven [36]. However, in our research, CNF apparently appears less cross-linked, resulting in a smaller surface area and pore volume. With the glycerol, the tensile properties decreased. As explained in many studies, the addition of plasticizer in biopolymers reduces crystallinity, which leads to a significant decrease in film strength and modulus [38,39,40]. In our case, the addition of glycerol also reduced the flexibility of the films, which is in contrast to previous investigations. The combination of decreasing tensile strength and strain at break is surprising, and the explanation could be the reduced density. The addition of glycerol lowered the density of the film (1.29 g∙cm^−3^). In the CNF films described in the literature, the film density was about 1.52 g∙cm^−3^ [37].

### 3.2. Water Contact Angle

**Figure 2:**
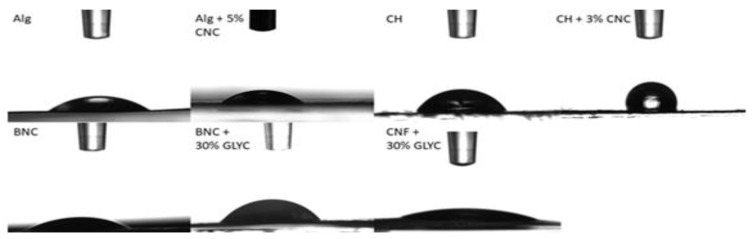
Visualization of the water contact angle for the selected films are shown in Table 1 (ALG—alginate; Alg + 5% CNC—alginate + 5% CNC; CH—chitosan; CH + 3% CNC—chitosan + 3% CNC; BNC—BNC; BNC + 30% GLYC—BNC + 30% glycerol; CNF + 30% GLYC—CNF + 30% glycerol).

The information about interactions between films and water is very important for packaging. Hydrophobic or hydrophilic character is frequently determined by surface free energy and surface morphology. The contact angle of the surface with water is important to characterize a material as such and can give an impression of absorption and adhesion as well. A lower contact angle with water is an indicator that films are hydrophilic and hygroscopic. The most wettable surfaces have low values (˂20°) and the hydrophobic surfaces have high values of contact angle (˃90°) [38]. In Table 1 and Figure 1, contact angles of polysaccharide films with water are recorded. Comparing films consisting of only one biopolymer, chitosan exhibited the highest contact angle (75°) and CNF with the addition of 30% glycerol the lowest (23°) (Figure 2). Alginate and nanocellulose films can be considered as hydrophilic. When combining chitosan and alginate with CNC, the contact angle with water increased by 44% and 49%, respectively. This trend was also confirmed by Mao et al. [34], where the hydrophobicity of chitosan/CNC film increased compared to only chitosan film. Although cellulose consists of β-D-glucopyranose units with three hydroxyl groups, which are responsible for the hydrophilic character of cellulose, electrostatic association and hydrogen bonding bids the CNC and chitosan molecules closely together, which improves the hydrophobicity [34,39,40,41]. The higher contact angle can also be a result of changed morphology, which interrupts water spreading. CNF + 30% glycerol films are the most hydrophilic. This behaviour is an indication of the high affinity of glycerol for water. Glycerol in cellulose films tends to migrate to the surface, as also confirmed by Spoljaric et al. [42]. However, this was not the case for BNC films, where the addition of glycerol led to an increase (by 141%) in the contact angle. It is possible that glycerol filled the pores between the fibres, reducing porosity, and thus decreased the surface free energy. In packaging, more hydrophobic materials are generally desired as they offer a wider range of applications [40].

### 3.3. Barrier Properties of the Films

The gas phase permeation through a non-porous material occurs by adsorption at the front interface, diffusion through the material, and desorption at the rear interface, and is often measured with transfer rate, permeance, and permeability. The transmission rate is the volume or weight of a permeating agent (e.g., oxygen or moisture) passing through a film per unit surface area and time in equilibrium with the test conditions. 

The addition of CNC to alginate and chitosan reduced WVTR by 15% and 45%, respectively. OTR decreased by 45% for alginate and CNC and by 38% for chitosan and CNC, compared to pure film. It is obvious that the nanostructure of nanocomposites created a tortuous path for oxygen, which was also demonstrated by Enescu et al. [43].

As shown in the tensile properties of BNC with added glycerol, the water absorbed into the matrix of the films had a plasticizing effect, reducing tensile strength and increasing strain at break. In this area, adsorbed water molecules promoted the reorganization of the polymer chains, which was reflected in the change in water permeability in this area. The water barrier properties decreased enormously by 198%. The same trend was observed for oxygen permeability, which decreased for 77%.

For CNF film with added glycerol, it was impossible to measure the WVTR because the sample was too fragile and, therefore, this test was not performed. On the other hand, the OTR results of the sample showed that the oxygen permeability increased enormously, compared to pure CNF from the previous research [44]. This could be the reason for the microcracks that were present on the sample because the fibrils were very short, the film was fragile, and the oxygen could easily pass through.

**Figure 3:**
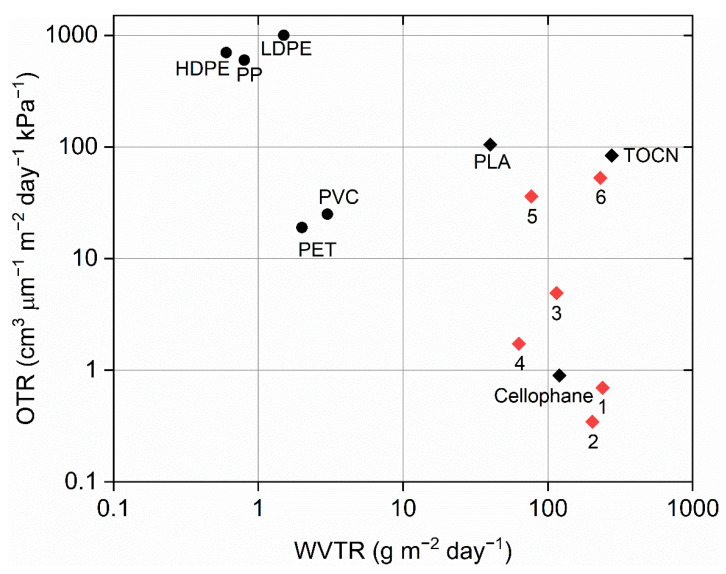
WVTR and Oxygen transmission rate (OTR) of some synthetic polymers compared to our nanocomposites packaging films. Adapted from [45] (Black circles—Petroleum-based Polymers, Black diamonds—commercial Biopolymers, red diamond—biopolymer nanocomposites tested in this study) (HDPE—high-density polyethylene; LDPE—low-density polyethylene; PP—polypropylene; PVC—polyvinyl chloride; PET—polyethylene terephthalate; PLA—polylactic acid; TOCN—TEMPO-oxidized cellulose nanofibers).

In Figure 3, the results of WVTR and OTR of the films fabricated in this study as well as for other commercially polymer blends for comparison are presented. Pure polysaccharide films had higher WVTR compared to most commercial packaging films, except TOCN (TEMPO-oxidized cellulose nanofibers). When CNC was combined with alginate and chitosan, the OTR results showed higher OTR compared to cellophane but still lower OTR compared to bio-based polylactic acid films. Pure BNC and BNC with added glycerol showed similar oxygen and water vapor permeability to PLA and TOCN. 

The addition of CNC reduced the WVP value of alginate films from 9.36 g/(m∙s∙Pa) to 7.32 g/(m∙s∙Pa) (Table 2). An even greater decrease in this value was observed with 3% CNC addition into the films made of chitosan. In this case, the value dropped from 10.1 g/(m∙s∙Pa) to 1.39 g/(m∙s∙Pa). The addition of glycerol drastically increased the WVP value of BNC films (from 3.62 g/(m∙s∙Pa) to 9.17 g/(m∙s∙Pa).

### 3.4. Visualization and Morphology of the Films

**Figure 4:**
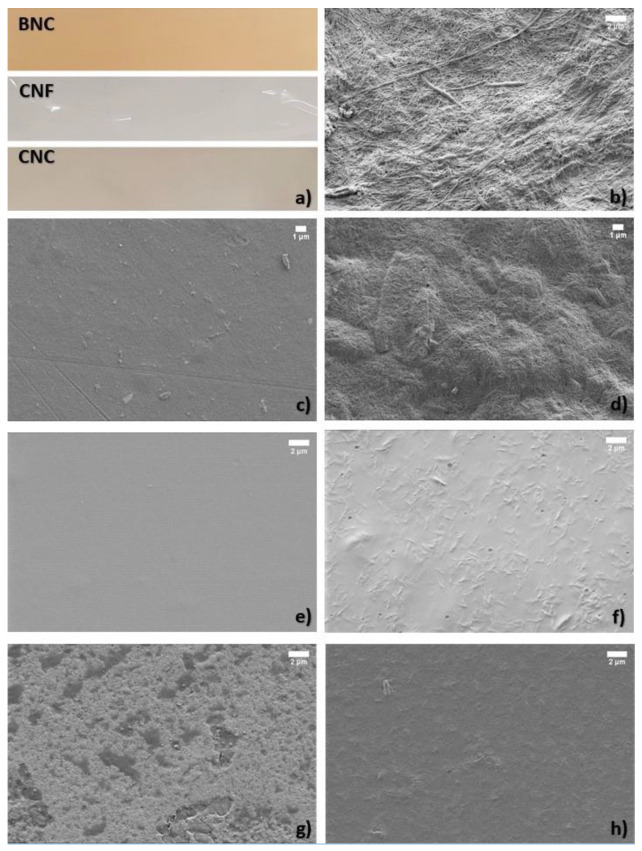
(**a**) Visualisation of BNC, CNF, and CNC films. SEM micrographs of (**b**) BNC, (**c**) CNF film with 30 wt% glycerol, (**d**) CNC film with 30 wt% glycerol, (**e**) chitosan film with 30 wt% glycerol, (**g**) alginate + 5% CNC, (**f**) chitosan + 3% CNC, and (**h**) alginate film with 30 wt% glycerol.

CNF films appeared as fully transparent materials with a glossy surface, CNC films were slightly whitish and pale semi-transparent material, while BNC films were slightly brownish semi-transparent material (Figure 4a). CNC additive in amounts of 3–5% *w/w* did not change the transparency or colour of chitosan or alginate films. CNC and CNF have been known for their application in optically transparent films [46]; however, CNC can be less transparent and haze depending on the size of crystals and thickness of film [47]. The brownish colour of BNC films can be explained by the influence of fermentation medium, which was apple juice and culturing bacteria used for obtaining the initial material, since the growing conditions have a significant effect on the properties of bacterial cellulose [48].

Inspection of the films with SEM revealed that BNC and CNF were in the shape of a long, fibrillary network (Figure 4b,c) with an average diameter of fibril of 69 ± 24.3 nm and 26 ± 6.5 nm, respectively. On the other hand, CNC were rod-like shaped particles with an average width of 83 ± 18.8 nm and length of 777 ± 112 nm (Figure 4d). Chitosan and alginate films have a smooth surface before being mixed with nanocellulose (Figure 4e,g, respectively). In alginate-CNC composite, clusters of CNCs are visible, indicating that although both alginate and CNCs are hydrophilic, CNC tends to agglomerate when mixed into alginate matrix (Figure 4g), while in chitosan film, the CNCs are more homogeneously dispersed (Figure 4f).

### 3.5. Film Density and Moisture Adsorption

Alginate and chitosan films have the highest density, and it decreases with the addition of nanocellulose, which corresponds to observations on the films’ morphology (Figure 4d,e). The mixing of CNC into the alginate or chitosan matrix creates porosity, which is responsible for the lower density. Wang et al. [44] described that CNC has a “rice-like” structure, which causes the changes in the microstructure of the films and consequently in the density.

Similarly, BNC and CNF have lower density compared to films with chitosan and alginate matrix but can be slightly increased with the addition of glycerol (Table 2). 

As it was said earlier in section about contact angle measurements, interactions between films and water are significant for packaging and hydrophobicity or hydrophilicity properties of films can determine their application areas. Moisture absorption from air medium was tested and all biopolymer films, regardless of composition, demonstrated hygroscopic behaviour at high (85%) relative humidity, therefore showing their ability to absorb water vapour from ambient air. Such a high RH number was chosen in order to investigate the variation of absorption between films of different compositions. Although CNC film was not selected for detailed investigation because of cracks and breakage, the moisture absorption was measured and the comparison of nanocellulose films demonstrated moisture absorption of 13.3 ± 0.2% in the case of CNC and slightly higher in the cases of CNF (20.4 ± 0.6%) and BNC (21.3 ± 1.0%). Fibrillated forms of nanocellulose tend to absorb more water due to the fibrillar structure and bigger proportion of amorphous regions, where it is easier for water molecule to get into and to bond with hydroxyl groups of cellulose. After 24h in high humidity, nanocellulose films became more flexible on touch, especially BNC one; however, they retain their shape and part of their stiffness. Cellulosic fibres, being hydrophilic in nature, absorb moisture from their environment until equilibrium is reached [49]. Adding plasticizer to BNC increased moisture absorption by 120%, improving the highly hygroscopic behaviour of glycerol.

Chitosan and alginate films absorb more moisture than pure nanocellulose films. It is 49.2 ± 1.5% in the case of pure chitosan and 57.4 ± 1.6% in the case of pure alginate. It is worth noting that moisture changes the structure of chitosan and alginate films—they become sticky, loose, lose their shape and stiffness, and stick to the surfaces. However, the addition of 3–5% CNC decreased the moisture absorption of films. Moisture absorption values decreased to different extents depending on the amount of nanofillers. The addition of 3% CNC decreased the moisture absorption of chitosan films by 15.1%, however, in the case of alginate, the addition of 5% nanocellulose decreased the moisture absorption by 10.8%. Therefore, it can be concluded that nanocellulose additives help to preserve the structure of chitosan and alginate films in a humid environment and should be considered. Ability of NC to prevent the absorption of moisture of NC-reinforced chitosan films were investigated elsewhere [30].

## 4. Conclusions

In this research, 25 different formulations of 5 sustainable biopolymers were used to produce and characterize thin and flexible films with potential use for packaging purposes. Results of mechanical testing showed that the addition of 3–5% CNC, CNF, and BNC improved the tensile strength of chitosan films, however, for alginate films, the impact of NC depended on the type and amount of additive and was positive only when CNC was added. From the cellulose-based films, BNC had the highest tensile strength, 60 ± 11 MPa and 53 ± 5 MPa without and with glycerol accordingly, CNF (with glycerol) followed with a result of 47 ± 3 MPa. CNC films appeared as slightly whitish and pale semi-transparent material, while BNC films were slightly brownish semi-transparent material, and other formulations were fully transparent. Seven formulations—alginate, alginate +5% CNC, chitosan, chitosan +3% CNC, BNC with and without glycerol, and CNF with glycerol—were selected as the most appropriate for packaging purposes based on visual/physical appearance and mechanical properties of films, and characterized in terms of morphological examination with SEM, density, contact angle, surface energy, water absorption, and oxygen and water barrier properties. SEM examination of cellulose-based films revealed typical morphology of crystalline and fibrillar forms of NC. Investigation of mixed formulations revealed more homogenous dispersing of CNC in chitosan than in alginate. Alginate and chitosan films had the highest density, which decreased with the addition of CNC because of greater porosity. Water contact angle differed among selected samples, the lowest was detected for CNF with glycerol (23 ± 1) and the highest for chitosan with 3% CNC (108 ± 2), the other films having results in the range from 39° to 75° and showing the increase of hydrophobicity of chitosan and alginate with the addition of CNC. This fact was approved also by moisture absorption results, which showed reduced moisture absorption for chitosan and alginate films after the addition of CNC for 15.1% and 8.8%, accordingly. Overall, chitosan and alginate films absorb more moisture than pure nanocellulose films, however, the addition of CNC can help to preserve the structure and function of chitosan and alginate packaging materials in humid environments. Therefore, materials with higher hydrophobicity would be more appropriate as they offer a wider range of applications. Results of barrier properties showed that the addition of CNC improved the WVTR of alginate by 15% and of chitosan films by 45%, while OTR decreased by 45% for alginate with CNC and by 38% for chitosan with CNC, compared to one component films. The addition of glycerol to BNC films decreased WVTR almost twice and OTR for 77%. 

Based on the findings of this study, it was concluded that polysaccharide-based films with added CNC are the most suitable for packaging purposes. With good oxygen barrier, water barrier that is comparable to PLA, and good mechanical properties, we propose that such films would be a good alternative to conventional plastic packaging used for ready-to-eat foods with short storage time, such as sandwiches and solid, refrigerated vegetables (for instance cucumbers, cauliflower, broccoli).

## Figures and Tables

**Table 1:** Selected polysaccharide films are based on the specified properties and include the most promising candidate from each biopolymer. These films are also subjected to a full characterization of morphological and barrier properties (values are given as mean ± SD).

Sample Name	Film Composition	Thickness (µm)	Tensile Strength (MPa)	Strain at Break (%)	Water Contact Angle (°)
Film 1	Alginate	55 ± 3	40 ± 10	22 ± 6	39 ± 2
Film 2	Alginate + 5% CNC	50 ± 2	43 ± 4	28 ± 4	58 ± 3
Film 3	Chitosan	123 ± 3	14 ± 2	51 ± 4	75 ± 3
Film 4	Chitosan + 3% CNC	70 ± 1	31 ± 2	55 ± 6	108 ± 2
Film 5	BNC	66.4 ± 0.8	60 ± 11	4.2 ± 1	46 ± 3
Film 6	BNC + 30% glycerol	56 ± 2	53 ± 5	10 ± 0.4	65 ± 4
Film 7	CNF + 30% glycerol	31 ± 3	47 ± 3	2.4 ± 0.6	23 ± 1

**Table 2:** Density, moisture absorption, water vapor transmission rate (WVTR), properties of films (values are presented as mean ± SD). Water vapor permeability results (WVP) are presented as a calculated mean value.

Sample Name	Film Composition	Film Density (g∙cm^−3^)	Moisture Absorption (%)	WVTR (g∙cm^−1^∙day^−1^)	WVP (g/(m∙s∙Pa))
Film 1	Alginate	1.87 ± 0.2	57.4 ± 1.6	239 ± 8	9.36
Film 2	Alginate + 5% CNC	1.34 ± 0.16	51.2 ± 1.9	203 ± 5	7.23
Film 3	Chitosan	2.00 ± 0.35	49.2 ± 1.5	115 ± 9	10.07
Film 4	Chitosan + 3% CNC	1.05 ± 0.10	36.5 ± 1.8	63 ± 2	1.39
Film 5	BNC	0.74 ± 0.05	9.7 ± 0.8	77 ± 4	3.62
Film 6	BNC + 30% glycerol	0.79 ± 0.10	21.3 ± 1.0	230 ± 11	9.17
Film 7	CNF + 30% glycerol	1.29 ± 0.20	20.4 ± 0.6	Not applicable	Not applicable

## Data Availability

Data is contained within the article and Supplementary Material.

## Supplementary Materials

The following are available online at https://www.mdpi.com/article/10.3390/polym13152523/s1, Table S1: Overview of all the prepared biopolymers films and nanocomposites together with evaluated mechanical properties and moisture absorption.
