# Supplementary material for: Functional Nanocellulose, Alginate and Chitosan Nanocomposites Designed as Active Film Packaging Materials
Source: Polymers (Basel). 2021 Jul 30;13(15):2523. doi: 10.3390/polym13152523 (PMC8348297; doi:10.3390/polym13152523)
Supplement: Supplementary file 1 [file polymers-13-02523-s001.zip › polymers-1279718-supplementary.pdf]

Table S1. Overview of all the prepared biopolymers films and nanocomposites together with evaluated mechanical properties and moisture absorption.

	Film	Tensile strength [MPa]	Strain at tensile strength [%]	Moisture absorption [%]
1	Alginate	37.9 ± 9.5	21.7 ± 6.2	57.4 ± 1.6
2	Alginate + 3 % CNF	11.6 ± 1.3	8.0 ± 3.4	-
3	Alginate + 5 % CNF	11.7 ± 0.7	15.5 ± 3.6	51.2 ± 1.9
4	Alginate + 3 % CNC	41.3 ± 3.6	14.6 ± 2.0	52.9 ± 2.7
5	Alginate + 5 % CNC	42.6 ± 3.6	28.2 ± 4.1	-
6	Alginate + 3 % BNC	38.7 ± 3.8	17.4 ± 1.3	51.8 ± 3.4
7	Alginate + 5 % BNC	31.2 ± 2.1	14.5 ± 2.9	-
8	Alginate + 3 % MIX	15.4 ±1.9	17.2 ± 3.0	-
9	Alginate + 3 % (CNF+CNC)	15.8 ± 2.4	19.9 ± 4.4	-
10	BNC	60.1 ± 10.7	4.2 ± 1.2	9.7 ± 0.8
11	BNC + 30 glyc	53.1 ± 4.5	10.3 ±0.4	21.3 ± 1.0
12	CNF	-	-	-
13	CNF + 30 glyc	47.0 ± 3.4	2.4 ± 0.6	20.4 ± 0.6
14	Chitosan	14.0 ± 2.2	51.2 ± 3.5	49.2 ± 1.5
15	Chitosan + 3 % CNF	16.3 ± 2.2	57.4 ± 3.6	46.1 ± 3.8
16	Chitosan + 5 % CNF	23.8 ± 2.7	50.9 ± 2.9	43.7 ± 2.0
17	Chitosan + 3 % CNC	30.9 ± 2.2	54.5 ± 5.8	36.5 ± 1.8

18	Chitosan + 5 % CNC	29.7 ± 2.1	51.0 ± 5.4	39.3 ± 2.3
19	Chitosan + 3 % BNC	22.6 ± 1.2	42.5 ± 2.6	46.6 ± 1.3
20	Chitosan + 5 % BNC	23.8 ±2.1	38.1 ± 3.4	42.4 ± 4.6
21	Chitosan + 3 % (CNF+CNC+BNC)	29.0 ±3.3	50.5 ± 3.1	46.1 ± 1.3
22	Chitosan + 3 % (CNF+CNC)	26.1 ± 4.1	51.7 ± 8.2	43.5 ± 3.0
23	CNF+BNC+CNC+glyc	32.8 ± 3.3	14.4 ± 1.1	17.3 ± 2.9
24	75CNF+25BNC+glyc	37.8 ± 3.5	9.5 ± 1.0	24.2 ± 2.0
25	CNC	-	-	13.3 ± 0.2
